# Editorial: Plant natural resins: from formation mechanism to ecological significance

**DOI:** 10.3389/fpls.2024.1412805

**Published:** 2024-04-30

**Authors:** Xupo Ding, Jinhui Chen, Petr Maděra

**Affiliations:** ^1^ Guangxi Key Laboratory for Polysaccharide Materials and Modifications, School of Marine Sciences and Biotechnology, Guangxi Minzu University, Nanning, China; ^2^ School of Breeding and Multiplication (Sanya Institute of Breeding and Multiplication), Hainan University, Sanya, China; ^3^ Department of Forest Botany, Dendrology and Geobiocoenology, Faculty of Forestry and Wood Technology, Mendel University in Brno, Brno, Czechia

**Keywords:** plant resins, formation mechanism, ecological significance, evolution, (A)biotic stress

In nature, only relatively old original plants can produce a small amount of natural resin, and the harvest of resins is invariably destructive to the original plants, resulting in the rarity of plant resins (Ding et al., 2020; Maděra et al., 2020). Modern natural product chemistry indicates that plant resins contain various kinds of terpenes, saponins, flavonoids, steroids, and their derivatives as their main aromatic or pharmacodynamic compounds (Cao et al., 2019; Li et al., 2021). Owing to their economic and medicinal importance arising from their use as spices and in medicinal products, the original wild plants of natural resins have been exploited excessively, with many of these species considered endangered on the IUCN Red List. It is necessary to investigate their genetic background to explore the mechanisms underlying resin formation and develop induction technologies to provide a theoretical or technological foundation for ensuring sustainable production of natural resins from endangered species. However, the genetics of the original plants and the mechanisms underlying the formation of plant resins remain poorly understood. Specifically, it remains to be elucidated how the original plant accumulates diversiform compounds in its resin, of which a few may be deleterious to the original plant itself, which may imply the ecological significance of plant resins.

In this context, this edition on natural plant resins presents a total of seven articles with six original research articles and one review article covering studies on natural plant resins, such as agarwood, rosin, dragon’s blood, and myrrh resins produced by *Aquilaria agallocha*, *Aquilaria sinensis*, *Commiphora wightii*, *Dracaena draco*, *Larix decidua* and *Pinus pinaster*. These seven manuscripts address various aspects of research related to natural plant resins, including the dynamics of the formation of these resins and the underlying regulation mechanisms, the activities of the main compounds, and interactions with plant microorganisms.

Agarwood is a popular spice and traditional medicine (Ding et al., 2020), but the regulatory mechanisms underlying its formation remain unclear, although previous studies have suggested the involvement of endophytic microorganisms. Li et al. found that a longer than usual induction time boosts Qi-Nan agarwood yield, but the content of alcohol extracted per unit weight and the diversity of endophytic fungal communities decreased in this high-yield Qi-Nan agarwood. Moreover, the sesquiterpene content increased, whereas that of chromones decreased in this Qi-Nan agarwood. However, correlation analysis revealed a significant positive correlation between endophytic fungi and the yield, alcohol extract content, sesquiterpene content, and chromone content of Qi-Nan agarwood, suggesting that endophytic fungi promote Qi-Nan agarwood formation. Microorganism infection occurs alongside reactive oxygen species (ROS) bursts in plants, and the respiratory burst oxidase homolog (*Rboh*) gene family is implicated in this process. Begum et al. demonstrate that ROS bursts occur during agarwood formation and have identified 14 *Rboh* genes in *A. sinensis* and *A. agallocha*. Two of these, *AaRbohA* and *AaRbohC*, facilitate *A. agallocha* plants against (a)biotic stress. The 2-(2-phenethyl) chromone and its derivatives are the biomarkers for agarwood formation. Zhang et al. explored the molecular evolution of the basic leucine zipper (*bZIP*) gene family in Malvales and found that the number of *bZIP* genes in the *A. sinensis* genome is lesser than that in other species that have undergone two whole genome duplications in Malvales. Moreover, *AbZIP14* and *AsbZIP41*, a pair of paralogous genes in the *A. sinensis* genome, were both highly expressed during agarwood formation but were subject to a differential regulatory mechanism via interactions with polyketide synthases, the key enzymes in chromone biosynthesis.

Rosin, a resin derived from the steam distillation of oleoresin, constitutes approximately 95% of the weight of oleoresin and serves as a sustainable industrial raw material. It is used in various products such as varnishes, chewing gums, emulsifiers, polymers, and coatings (Neis et al., 2019). The two manuscripts focusing on this resin suggest that the contents of the essential oils in this resin and its main compounds correlate with the harvest time and manufacturing process using gas chromatography–mass spectrometry (GC–MS) or nuclear magnetic resonance spectroscopy. Batista et al. suggest that standardization and storage, including the registration of the collection data and geographic location, are necessary to guarantee a reproducible chemical composition in production to avoid adulteration by volatile organic compounds from other plants in the manufacturing process. They also suggest that some adulterants or compounds (*trans*-carveol) are due to the changes associated with the aging of original plants. Another study by Pinheiro et al. indicates that resin acid content in Portuguese rosin is independent of sample periods and locations and shows no significant correction with edaphoclimatic parameters. These results suggest that the Portuguese rosin harvested from *P. pinaster* is characterized by high abietane content and stable chemistry.

Natural plant resins are actively used in modern pharmaceuticals (Shuaib et al., 2013; Seyfullah et al., 2018). Patel et al. demonstrate that the hexane extraction of gum resin from *C. wightii* exhibits significant larvicidal activity against the mosquito *Aedes aegypti*, which causes lethal dengue fever in tropical areas. A series of terpenes have been identified from the gum using GC–MS, and these monomeric compounds possess potential inhibitory activities against *γ*-aminobutyric acid receptors, octopamine receptors, and acetylcholinesterase. Li et al. also indicated that Qi-Nan agarwood exhibits significant free radical and ROS scavenging activities.

Resins have invariably been considered the defense metabolites of plants against (a)biotic factors (Ding et al., 2018). However, the processes involved in their secretion at the cellular level in plants have not been elucidated, particularly for species without resin ducts or canals, e.g., *Dracaena* or *Aquilaria* spp. Tulik and Jura-Morawiec provide an overview of the morpho-anatomical characteristics of the different types of secretory cells for resin release by focusing on their location, origin, formation mechanism, and ecological importance and suggest that parenchyma cells may be involved in resin synthesis and secretion. Li et al. used plant anatomy and histochemistry techniques to demonstrate that starch and soluble sugars are consumed, and subsequently, agarwood is formed and filled in the ray cells and interxylary phloem of *A. sinensis*.

In the plant kingdom, approximately 10% of the species secrete resin, most of which are found in the tropics. Agarwood, dragon’s blood, frankincense, myrrh, and rosin have been mentioned in the history of many cultures as precious and scarce resources used as spices, preservatives, dyes, and traditional medicine since ancient times. These natural resins usually present application value in industry and medicine, and their original plants also present unique traits that have not been explored extensively until now. The articles included in this edition present the latest advances in this field, which will help strengthen the theoretical and technological foundation for using endangered plants to efficiently produce natural resins and inspire more scientists to contribute to this field.

## Author contributions

XD: Writing – original draft. JC: Writing – review & editing. PM: Writing – review & editing.
